# Large scale tissue histopathology image classification, segmentation, and visualization via deep convolutional activation features

**DOI:** 10.1186/s12859-017-1685-x

**Published:** 2017-05-26

**Authors:** Yan Xu, Zhipeng Jia, Liang-Bo Wang, Yuqing Ai, Fang Zhang, Maode Lai, Eric I-Chao Chang

**Affiliations:** 1 State Key Laboratory of Software Development Environment and Key Laboratory of Biomechanics and Mechanobiology of Ministry of Education and Research Institute of Beihang University in Shenzhen, Beijing, China; 2 Microsoft Research, Beijing, China; 3 Institute for Interdisciplinary Information Sciences, Tsinghua University, Beijing, China; 4 Graduate Institute of Biomedical Electronics and Bioinformatics, National Taiwan University, Taipei, Taiwan; 5 Department of Pathology, School of Medicine, Zhejiang University, Hangzhou, China

**Keywords:** Deep convolution activation feature, Deep learning, Feature learning, Segmentation, Classification

## Abstract

**Background:**

Histopathology image analysis is a gold standard for cancer recognition and diagnosis. Automatic analysis of histopathology images can help pathologists diagnose tumor and cancer subtypes, alleviating the workload of pathologists. There are two basic types of tasks in digital histopathology image analysis: image classification and image segmentation. Typical problems with histopathology images that hamper automatic analysis include complex clinical representations, limited quantities of training images in a dataset, and the extremely large size of singular images (usually up to gigapixels). The property of extremely large size for a single image also makes a histopathology image dataset be considered large-scale, even if the number of images in the dataset is limited.

**Results:**

In this paper, we propose leveraging deep convolutional neural network (CNN) activation features to perform classification, segmentation and visualization in large-scale tissue histopathology images. Our framework transfers features extracted from CNNs trained by a large natural image database, ImageNet, to histopathology images. We also explore the characteristics of CNN features by visualizing the response of individual neuron components in the last hidden layer. Some of these characteristics reveal biological insights that have been verified by pathologists. According to our experiments, the framework proposed has shown state-of-the-art performance on a brain tumor dataset from the MICCAI 2014 Brain Tumor Digital Pathology Challenge and a colon cancer histopathology image dataset.

**Conclusions:**

The framework proposed is a simple, efficient and effective system for histopathology image automatic analysis. We successfully transfer ImageNet knowledge as deep convolutional activation features to the classification and segmentation of histopathology images with little training data. CNN features are significantly more powerful than expert-designed features.

## Background

Histopathology image analysis is a gold standard for cancer recognition and diagnosis [1, 2]. Digital histopathology image analysis can help pathologists diagnose tumor and cancer subtypes, and alleviate the workload of pathologists. There are two basic types of tasks in digital histopathology image analysis: image classification and image segmentation. In the classification task, the algorithm takes a whole slide histopathology image as input, and outputs the label of the input image. Possible labels are pre-defined, and they can be certain types of cancer or normal. In segmentation, the algorithm takes part of a histopathology image as input, and segments the region in the input image with certain characteristics. In both tasks, a set of training data with ground truth labels and annotations is given. In this paper, we develop a common framework for all these relevant histopathology problems such as classification and segmentation, and a visualization approach to explore the characteristics of deep convolutional activation features which reveal key biological insights.

There are 3 main challenges in automatic analysis of digital histopathology images: the complexity of the clinical feature representation, the insufficient number of training images, and the extremely large size of a single histopathology image.

The first challenge reflects the difficulty in representing complicated clinical features. Feature representation plays an important role in medical image analysis [3, 4]. Histopathology of different cancer types can exhibit dramatically diverse morphology, scale, texture and color distributions, which makes it difficult to find a general pattern for tumor detection that can be applied to both brain and colon cancer. Therefore, feature representation [5] is very important in high-level medical image tasks such as classification and segmentation. Many previous works have focused on feature design, such as object-like [6, 7] and texture features [8, 9]. However, the specificity of their designs limits the application to a fixed image source.

Another major concern is the insufficient amount of training data in the medical images domain. The fact that a medical image dataset usually has a much smaller size than a natural scene image dataset makes the direct application of many previous machine learning algorithms inappropriate for medical image datasets. Two factors make collecting medical images costly. One is the low incidence of the studied disease. The low frequency of the studied disease has made the collection process harder since the number of images depends on the number of disease incidences. The other is the extensive amount of demanded labor for manual data annotation, since detailed manual annotation of medical images usually requires a great deal of effort. Moreover, since many clinical clues are hard to quantify, manual annotation is also intrinsically ambiguous, even if labeled by clinical experts.

Last problem, the enormous size of individual histopathology images, makes the histopathology image dataset considered large-scale; and increases the computation complexity, thus making image analysis more challenging. One typical whole histopathology section can be scanned to yield an image of a size larger than 100,000×100,000 pixels and containing more than 1 million descriptive objects. Usually, 12 to 20 scanned images will be made for each patient under the pathological section process. Due to the inherent large-scale property of a histopathology image dataset, the feature extraction model needs to be both time and memory efficient, and the learning algorithm should be designed to be able to extract as much information as possible from these large images.

Problems mentioned above exist in all tasks of automatic histopathology image analysis. Beyond that, classification and segmentation tasks also face some specific challenges. In classification, subtle distinctions between different cancer sub-types require features to be highly expressive. And the fact of unbalanced instances of different sub-types also handicap the classifiers. In the segmentation task, the definition of regions need to be segmented might be opaque, which makes the ground truth annotated by multiple pathologists slightly different. This ambiguity property becomes a challenge in the design of segmentation frameworks.

With the advent of deep convolutional neural network (CNN), CNN activation features have recently achieved tremendous successes in computer vision [10–16]. The emergence of large visual databases such as ImageNet, including more than 10 million images and more than 20,000 classes [17], enables CNNs to provide rich and diverse feature description from general images. Responses of CNN hidden layers provide different levels of image abstraction and can be used to extract complex features like human faces and natural scenes. It makes extracting sufficient information from medical images possible. Therefore, in this paper, we study the potentials of ImageNet knowledge via deep convolutional activation to extract features for the classification and segmentation of histopathology images.

Although CNN itself is capable of image classification [14] and segmentation [18], the extremely large size of a single histopathology image makes it unrealistic to perform classification or segmentation with CNN directly. On the one hand, it is not practical to construct a CNN with a very large input size. On the other hand, downscaling the entire histopathology image to an acceptable size for CNN will lose too much detail information, which makes it impossible to recognize, even for pathologists. Based on this fact, both our classification and segmentation frameworks adopt a patch sampling technique to leverage CNN activation features of much smaller local patches, such that essential local details will be preserved. Different strategies are then adopted for final results. In the classification framework, feature pooling is used to construct features for all slide images. In the segmentation framework, classification is performed at the patch level and the results are used to construct image-wide segmentation. Smaller patch size and smoothing are used to make the boundaries more accurate.

In order to make CNN activation features more suitable for histopathology images, we also fine-tune the ImageNet model to learn more subtle and insightful features that capture complex clinical representatives. In our experiments, fine-tuned CNN models can reach better accuracy on both classification and segmentation tasks.

Moreover, we explore the characteristics of the CNN activation features by visualizing individual components of the 4096-dimensional feature vector in histopathology image classification. Heatmaps of patch confidence for each image and discriminative patches with individual neurons of the CNN activation features are computed. Heatmaps explain which patches or regions provide strong responses that make their image fall into the corresponding category, and patches that represent the individual neuron response help us understand what characteristics these responses have from the perspective of each classifier. Through this visualization analysis, we discover some relationships between clinical knowledge and our approach’s responses.

In this paper, we propose a simple, efficient, and effective method using CNN activation features applied to classification and segmentation of histopathology images. From the experiments, our framework achieves good performance in two dataset. The advantages of our framework include:

1. The ability to transfer powerful CNN features of ImageNet to histopathology images, which solves the problem of limited amount of training data in histopathology image datasets;
2. The adoption of patch sampling and pooling techniques to leverage local descriptive CNN features, which makes the whole framework scalable and efficient on extremely large whole slide histopathology images;
3. The unified framework on two different cancer types, which indicates the simplicity and effectiveness of our approach.

We make two contributions to the field of automatic analysis of histopathology images:

1. A general-purpose solution to histopathology problems on extremely large histopathology images, which proves effective and efficient on two different types of cancers;
2. A visualization strategy that reveals the features learned by our framework have biological insights and proves the capability of CNN activation features in representing complex clinical characteristics.

An earlier conference version of our approach was presented by Xu et al. [19]. In this paper, we further illustrate that: (1) the framework methods can be applied to analyzing tissue types other than brain tumor, such as colon cancer; (2) fine-tuned features based on the ImageNet model are added; (3) heatmaps are introduced to explore which patches or regions provide strong responses in one image in the classification task, accompanying the previous visualization of individual neural responses.

### Related work

In recent years, usage of digital histopathology has exhibited tremendous growth. Researchers have been attempting to replace optical microscope with digital histopathology as the primary tool used by pathologists. Various replacement approaches are studied in [20–23]. Under the trend of adopting digital histopathology, several competitions have been held to boost the tumor histopathology research community, including the ICPR 2012 Mitosis Detection Competition [24], the MICCAI 2013 Grand Challenge on Mitosis Detection [25], the MICCAI 2014 Brain Tumor Digital Pathology Challenge [26], and the MICCAI 2015 Gland Segmentation Challenge Contest [27]. Our proposed framework achieved first place results in both classification and segmentation at the MICCAI 2014 Brain Tumor Digital Pathology Challenge [28].

Feature representation design is a prominent direction relating to histopathology images. Manually designed features include fractal features [29], morphometric features [30], textural features [31], and object-like features [32]. Kalkan [33, 34] exploits textural and structural features from patch-level images and proposes a two-level classification scheme to distinguish between cancer and non-cancer in colon cancer. Chang [35] proposes sparse tissue morphometric features at various locations and scales to distinguish tumor, necrosis, and transition to necrosis for the GBM dataset and tumor, normal, and stromal for the KRIC dataset. Due to the large amount of data, Chang also uses spatial pyramid matching to represent multi scale features. Rashid [36] designs two special gland features to describe benign and malignant glands in prostatic adenocarcinoma. The two features are the number of nuclei layers and the ratio of the epithelial layer area to the lumen area. Song [37] transforms the images with learning-based filters to obtain more representative feature descriptors. Sparks [38] proposes a set of novel explicit shape features to distinguish subtle shape differences between prostate glands of intermediate Gleason grades on prostate cancer. Sos Agaian [39] introduces new features for tissue description such as hyper-complex wavelet analysis, quaternion color ratios, and modified local patterns.

However, the major issue with these approaches is the difficulty in choosing discriminant features to represent clinical characteristics. Study [40] has also shown that features learned by a two-layer network are more powerful than manually designed representations of histopathology images. Nayak [41] explores sparse feature learning utilizing the restricted Boltzmann machine (RBM) to describe histopathology features in clear cell kidney carcinoma (KIRC) and GBM. These studies have shown that feature learning is superior to special feature designs. But there is a universal challenge in feature learning that the amount of training data is limited in many cases. In our case, only a few training images are available for classification and segmentation.

Using deep CNN features as generic representations is a growing trend in many medical image tasks. Some publicly available deep CNN models are utilized to extract features: Caffe [42] is exploited in a number of works [10, 11, 42] and OverFeat [43] is used by [16]. These features are commonly used in classification and object detection tasks [10, 11, 16, 42]. However, these studies only focus on natural images.

Powerful CNN is not only capable of performing classification, but also able to learn features, and several studies directly utilize this property of CNN on histopathology image analysis. Ciresan [24] modifies a traditional CNN into a deep max-pooling CNN to detect mitosis in breast histology images. The detection problem is cast as pixel classification. Information from a patch centered on the pixel is used as context. Their approach has won the first place in the ICPR 2012 mitosis detection competition. The training set only includes 5 different biopsy H&S stained slides containing about 300 total mitosis events. Cruz-Roa [44] presents a novel deep learning architecture for automated basal cell carcinoma cancer detection. The training set contains 1,417 images from 308 regions of interest of skin histopathology slides. In contrast, ImageNet [17] is comprised of around 14 million images, which is much larger than datasets of histopathology images. Based on our survey on feature design and feature learning, we decided to adopt CNN features trained by ImageNet to describe discriminative textures in histopathology images of brain tumor and colon cancers.

Fine-tuning is an important step in CNN learning. It maintains the original network architecture and treats the trained CNN as an initialization. After fine-tuning training, the new model can learn more subtle representations to describe new targeted tasks. Ross [45] proposes object detection using fine-tuning to improve 10% points from 44.7% (R-CNN *fc*_7_) to 54.2% (R-CNN fine-tuned *fc*_7_) in the VOC 2007 test. Zhang [46] presents a fine-grained classification. The accuracy improves from 68.07% using pre-trained CNN features to 76.34% using fine-tuned features. These studies demonstrate that fine-tuning is effective and efficient. In our case, on the basis of pre-trained CNN features, we implement the fine-tuning step to learn more subtle representations for histopathology images.

In addition to feature representations, histopathology image analysis also involves classification schemes. Xu [47, 48] introduce a novel model called multiple clustered instance learning to perform histopathology cancer image classification, segmentation, and clustering. Furthermore, Xu [49] presents context-constrained multiple instance learning to adopt segmentation. Gorelick [50] proposes a two-stage AdaBoost-based classification. The first stage recognizes tissue components and the second stage uses the recognized tissue components to classify cancerous versus noncancerous, and high-grade versus low-grade cancer. Kandemir [51] introduces a probabilistic classifier that combines multiple instance learning and relational learning to classify cancerous versus noncancerous. The classifier exploits image-level information and alteration in cell formations under different cancer states. Kalkan [33] proposes a two-stage classification. The first stage classifies patches into possible categories (adenomatous, inflamed, cancer and normal). The second stage uses the results from the first stage as features. Finally a logistic linear classifier recognizes cancerous versus noncancerous. In our case, a linear SVM classifier is used in consideration of its simplicity and speediness.

In classification, the inputs used are usually the resized original image [14]. The extracted CNN features are directly used as the last features to classify categories. There are some different methods in [14]. Sharif Razavian et al. [16] extracts 16 patches that include an original image, five crops (four corners and one center of 4/9 of the original image area), and two rotations and their mirrors. The CNN features are extracted when the 16 patches are used as the inputs. After that, the authors [16] take the sum of all the responses of the last layer as the final features. Gong et al. [11] samples patches in multi-scale levels, with a stride of 32 pixels. Multi-scale orderless pooling of deep convolutional activation features are extracted. Then the authors [11] aggregate local patch responses via vectors of locally aggregated descriptions (VLAD) encoding. In our method, inspired by [52] and the observation that histopathology images are extremely large up to the gigapixel size of an image, we use patch samplings to generate many patches to protect detailed local information and use feature pooling to aggregate the patch-level CNN features into the last features.

Histopathology image analysis is used in a wide range of research. Khan [53] proposes a nonlinear mapping approach to normalize staining. Image-specific color deconvolution is applied to tackle color variation when different tissue preparation, stain reactivity, user or protocol, and scanners from different manufacturers are used. Zhu [54] proposes a novel batch-mode active learning method to solve the challenges of annotation in scalable histopathological image analysis. Feature selection and feature reduction schemes [38, 55] are also important steps in histopathology image analysis.

## Methods

### CNN architecture

AlexNet [14] is a simple and common deep convolutional neural networks and can still achieve competitive performances in classification compared with other kinds of networks. Therefore, AlexNet architecture is used in our case. The CNN model we use in this paper is shared by the CognitiveVision team at ImageNet LSVRC 2013 [13] and its architecture is described in Table 1. It is analogous to the one used in [14], but without the GPU split, since a single modern GPU has sufficient memory for the whole model. This model was trained on the entire ImageNet dataset. Thus it is little different from what the CognitiveVision team used at ILSVRC 2013. The code used for training and extracting features is based on [14]. In the training step, we use the data pre-processing and data augmentation methods introduced in [14], transforming input images of various resolutions into 224 ×224. During feature extraction, input image is resized to 224 ×224 pixels and fed to the network. The output of the fc2 layer is used as an extracted feature vector.

**Table 1:** The CNN architecture

Layer	Dimension	Kernel size	Stride	Details
input	224×224×3	-	-	RGB channels
conv1	55×55×96	11	4	-
pool1	27×27×96	3	2	Max pooling
conv2	27×27×256	5	1	-
pool2	13×13×256	3	2	Max pooling
conv3	13×13×384	3	1	-
conv4	13×13×384	3	1	-
conv5	13×13×256	3	1	-
pool3	6×6×256	3	2	Max pooling
fc1	4096	-	-	-
fc2	4096	-	-	-

### Classification framework

The enormous size of the histopathology images makes it imperitive to extract features locally. Hence, each histopathology image is divided into a set of overlapping square patches with a size of 336 ×336 pixels for 20 × magnification and 672 ×672 pixels for 40 × magnification scale (they are both 151,872 ×151,872 nm^2^). The patches form a rectangular grid with 64-pixel stride, i.e., distance between adjacent patches. To further reduce the number of patches, we discard patches with only a white background, whose RGB values of all pixels are greater than 200. All selected patches are then resized to 224 ×224 pixels and fed into the network to obtain 4096-dimensional CNN feature vectors. The final feature vector of an image is computed over *P*-norm pooling. *P*-norm pooling, also known as softmax pooling, amplifies signals from a few patches, which is computed by

1 $$ f_{P}(\mathbf{v}) = \left(\frac1N \sum\limits_{i=1}^{N}\mathbf{v}^{P}_{i}\right){\frac{1}{P}},  $$

where *N* is the number of patches for an image, and *v*_*i*_ is the *i*-th patch feature vector. In our framework, *P*=3 (3-norm pooling) is used.

Moreover, in order to form a subset of more discriminative features and to exclude redundant or irrelevant features, feature selection is used in binary classification. Features are selected based on the differences between positive and negative labels. The difference of the *k*-th feature *diff*_*k*_ is computed by

2 $$ \mathit{{diff}_{k}} = \left| \frac{1}{N_{\text{pos}}} \sum\limits_{i\in\text{pos}}{v_{i, k}} - \frac{1}{N_{\text{neg}}} \sum\limits_{i\in\text{neg}}{v_{i, k}}\right|,  $$

where *k*=1,…,4096, *N*_pos_, and *N*_neg_ are the number of positive and negative images in the training set, and *v*_*i, k*_ is the *k*-th dimensional feature of the *i*-th image. Feature components are then ranked from largest *diff*_*k*_ to smallest, and the top 100 feature components are selected. For multiclass classification, no feature selection is used.

**Fig. 1:**
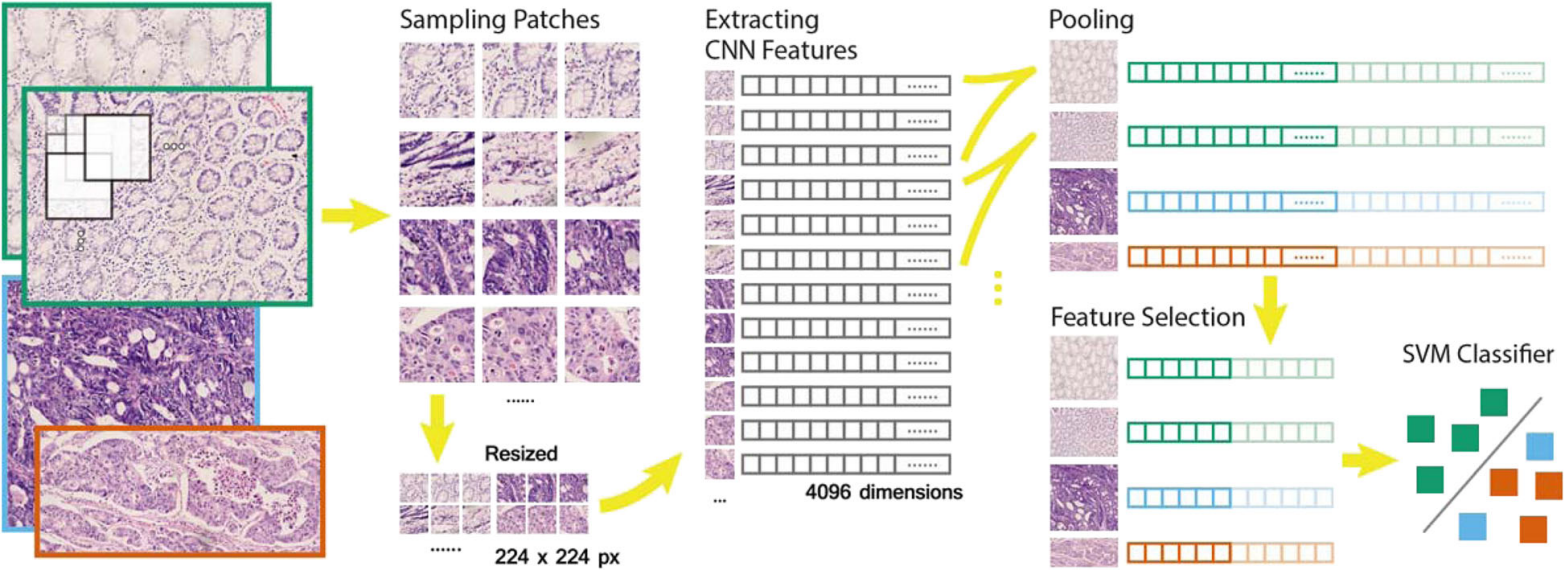
The classification workflow. First, square patches of 336 or 672 pixels in size are sampled on a rectangular grid, depending on the magnification scale of the image. Patches are then resized to 224 pixels in size as the input of our CNN model. A 4096-dimensional feature vector is extracted from the CNN model for each patch. A 100-dimensional feature is obtained by feature pooling and feature selection for each image. Finally, a linear SVM classifies the selected features. The figure shows a binary classification, where the positive (*blue* and *orange*) and negative (*green*) are GBM and LGG in brain tumor, cancer and normal in colon cancer respectively. In multiclass classification, a full feature vector of 4096 dimensions is used

Finally, a linear Support Vector Machine (SVM) is used. In multiclass classification, one-vs-rest classification is used. Figure 1 shows the workflow of our classification framework.

### Segmentation framework

**Fig. 2:**
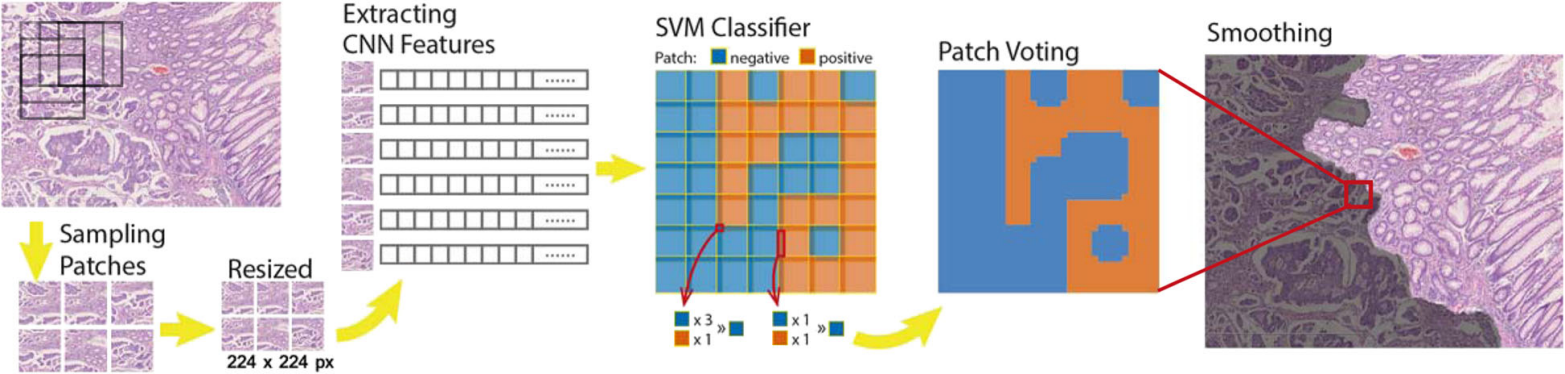
The segmentation workflow. Similar to classification workflow, square patches of 112 pixels in size are sampled on a rectangular grid with 8-pixel stride. Each patch is assigned a positive (*orange*) or negative (*blue*) label, which are necrosis vs. non-necrosis in brain tumor, and cancer vs. normal in colon cancer, respectively. In training phase, a patch is labelled positive if its overlap ratio with annotated segmented region is larger than 0.6. Patches are then resized and a 4096-dimensional feature vector is extracted from our CNN model. A linear SVM classifier is used to distinguish negative from positive patches. Probability mapping images are yielded utilizing all predicted confidence scores. After smoothing, positive segmentations are obtained

Medical image segmentation methods can be generally classified into three categories: supervised learning [29], weakly supervised [48] and unsupervised [32]. A supervised learning method can be used only if labelled data are available. Otherwise, other approaches (i.e. unsupervised methods) are needed. Since we have labelled training data, we propose a supervised learning framework for segmentation. In our framework, we reframe the segmentation problem as a classification one by performing classification on a collection of patches. Figure 2 illustrates the workflow of our segmentation framework.

Similar to the aforementioned classification framework, patches are sampled on a rectangular grid of 112 ×112 pixel patches in 8-pixel stride. 112 ×112 pixel patches are resized to 224 ×224 pixels to obtain their CNN feature vectors. A linear SVM is trained to classify all patches as positive or negative. Since a pixel can be covered by many overlapping patches with different labels, the final label for each pixel is decided by the majority vote of the patches covering this pixel. Since pixel-based voting provides many tiny positive or negative regions that lack biological meaning, we utilize several smoothing techniques to reduce region fractions. Small positive and negative regions with an area less than 5% of the full image size are removed.

In the MICCAI challenge, we further made two modifications to the training data for the final submitted model.

1. We observe that hemorrhage tissues appear in both non-necrosis and necrosis regions. Hence, we manually relabel hemorrhage patches in the necrosis regions as non-necrosis patches. This results in misclassification of hemorrhage patches at test time in the prediction stage, but since those patches are usually in the interior of the necrosis region, such errors can be corrected by the post-processing.
2. We observe that training images are non-uniform and have various sizes. Additionally, the training data is not evenly distributed. In the final model for submission, we augment the instances of missed regions and false regions generated by leave-one-out cross-validation on the training data.

### Dataset

We benchmark our classification framework and segmentation framework on two histopathology image datasets: the MICCAI 2014 Brain Tumor Digital Pathology Challenge and a colon cancer dataset. To illustrate the advantages of our frameworks, we also benchmark other approaches and other types of features on the same datasets.

For the MICCAI challenge [26], digital histopathology image data of brain tumors are provided by the organizers. In classification (sub-challenge I), the target is to distinguish images of glioblastoma multiforme (GBM) and low grade glioma (LGG) cancer. The training set has 22 LGG images and 23 GBM images, and the testing set has 40 images. In segmentation (sub-challenge II), the goal was to separate necrosis and non-necrosis regions from GBM histopathology images, since necrosis is a significant cue for distinguishing LGG from GBM. The training set included 35 images and the testing set includes 21 images. The image resolutions are either 502 nm/pixel or 226 nm/pixel, corresponding to 20 × and 40 × source lens magnification, respectively.

For colon cancer, H&E stained histopathology images are provided by the Department of Pathology of Zhejiang University in China and are scanned by the NanoZoomer slide scanner from Hamamatsu. Regions containing typical cancer subtype features are cropped and selected following the review process by three histopathologists, in which two pathologists independently provide their results and the third pathologist merge and resolve conflicts in their annotations. A total of 717 cropped regions were used as our dataset, with a maximum scale of 8.51 ×5.66 mm and average size 5.10 mm^2^. All images are of 40 × magnification scale, i.e. 226 nm/pixel. 355 cancer and 362 normal images are used for binary tasks. For multiclass classification, there are 362 normal (N), 154 adenocarcinoma (AC), 44 mucinous carcinoma (MC), 50 serrated carcinoma (SC), 38 papillary carcinoma (PC), and 45 cribriform comedo-type adenocarcinoma (CCTA) images (a total of 693 images that were used). 24 cancer images are disregarded in multiclass classification because there are too few instances in their cancer categories. Half of the images are selected as the training data and other images are used as testing data. The proportion of each cancer subtype in the testing data are the same as the full dataset. In the segmentation task, 150 training and 150 testing images are selected from the dataset. They are resized to a 10 × magnification scale (904 nm/pixel) and then cropped to 1,280 ×800 pixels. This is the same setting used in [32] for their algorithm GraphRLM. The segmentation ground truth of colon cancer images was annotated by pathologists, following the same review process mentioned before.

### Experiment settings

#### Classification

To illustrate the advantages of CNN features, we compare CNN features with manual features (that have fixed extraction algorithms) within our proposed framework. Only the feature extraction step in the framework is modified. In our experiments, generic object recognition features including SIFT, LBP, and L*a*b color histogram are adopted (following settings in [48]), concatenating into a total of 186 feature dimensions. This approach is denoted by **SVM-MF**, and our proposed framework using CNN features is denoted by **SVM-CNN**.

To show the effectiveness of patch sampling, we compare our framework with the approach that uses CNN features directly, without patch sampling. In this approach, the full histopathology image was resized to 224 ×224 pixels and fed to CNN to extract the image-level features. Then a linear SVM was used to perform classification. This approach is denoted by **SVM-IMG**.

Furthermore, we compare our classification framework with previous approaches Multiple Clustered Instance Learning (MCIL) [48] and Discriminative Data Transformation [37]. They are denoted by **MCIL** and **TRANS**, respectively. In **MCIL**, the patch extraction setting is the same as our approach. The softmax function here was the GM model and the weak classifier was the Gaussian function. The parameters of the algorithm are the same as described in the original study. In **TRANS**, learning-based filters are applied to original images and feature descriptors [37]. We follow settings in their original work (image filters of size *X*=3,5,7 and feature filter of size *Y*=5) and use a linear SVM as the classifier.

In all approaches a linear SVM (**SVM-IMG**, **SVM-MF**, **SVM-CNN** and **TRANS**), L2-regularized SVM with linear kernel function is adopted in experiments, whose cost function is $\frac {1}{2}w^{T} w+C\sum _{i=1}^{l}(\max (0,1-y_{i}w^{T}x_{i}))$. Open-source toolbox LIBLINEAR [56] is used to optimize SVM. The value of parameter *C* was chosen from {0.01,0.1,1,10,100} and the optimal value is determined by cross-validation on training data.

#### Segmentation

Similar to classification, we compare CNN features with manual features. Settings of manual features are the same as classification experiments. This approach is denoted by **SVM-MF**, and our proposed framework using CNN features is denoted by **SVM-CNN**.

To further improve segmentation results, the CNN model trained by ImageNet is fine-tuned on histopathology images to explore features more suitable for this task. In our experiments, we replace the CNN’s ImageNet-specific 1000-way classification layer with a randomly initialized 2-way classification layer. The CNN architecture remains unchanged. We start a stochastic gradient descent (SGD) at a learning rate of 0.0001. The learning rate is used in the unmodified layers, which is one tenth of the initial pre-training rate on ImageNet. We train the CNN model for 20 epochs, and the learning rate is not dropped during the training process. Besides features being extracted from the fine-tuned CNN model, other steps of the segmentation framework do not change. This approach is denoted by **SVM-FT**.

In addition, we compare our segmentation framework with a previous approach GraphRLM [32]. Since both ours and their original dataset are colon cancer datasets at same magnification scale, the parameters in our experiment are set the same as given in their publication: *r*_min_=8, *r*_strel_=2, *win*_size_=96, *dist*_thr_=1.25, and *comp*_thr_=100. This approach is denoted by **GraphRLM**.

The settings of linear SVM are the same as classification experiments.

### Evaluation

For classification tasks, accuracy is used as the evaluation score. For segmentation tasks, the evaluation follows the rule provided by the organizers of the MICCAI challenge, which computes the average of every image’s ratio of overlapping area size over a total involved area size of ground truth and results predicted by the algorithm. The computation of a score is as follows. A mapping defines a set of pixels of image *i* that are assigned to a positive label. Let the ground truth mapping of the segmentation of image *i* be *G*_*i*_ and the mapping generated by the algorithm be *P*_*i*_. The score for image *i*, *S*_*i*_, is computed as

3 $$ S_{i} = \frac{2\left|P_{i} \cap G_{i}\right|}{\left|P_{i} \cup G_{i}\right|},~i = 1,\ldots, K,  $$

where *K* is the number of total images. The evaluation score (called accuracy) is the average of *S*_*i*_.

For brain tumor tasks, since the organizers of the MICCAI challenge did not provide ground truth labels and annotations of testing data, we use 5-fold cross-validation for classification and leave-one-out cross-validation for segmentation in our experiments. Also, the modifications mentioned in Section 2.3 do not apply in our own cross-validation experiments.

## Results and discussion

### Classification results

In the MICCAI challenge, our final submission of classification task achieved 97.5% accuracy on the testing data, ranking first place among other participants. Table 2 shows the results of some of the top-performing methods provided on the submission website [28]. Our results are satisfying and the difference between our performance and the second-place team’s is up to 7.5%, which proves that our method can achieve state-of-art accuracy, even given a relatively small data size, with the help of ImageNet.

**Table 2:** Classification performance in the MICCAI challenge

	Accuracy	Place
Anne Martel	75.0%	4th
Hang Chang [30]	85.0%	3rd
Jocelyn Barker	90.0%	2nd
Our method [19]	97.5%	1st

We compare our method with state-of-art methods in training data from the MICCAI challenge. Table 3 summarizes the performances of some of state-of-art approaches. Our results are good compared with other methods. The method [57] uses two-stage, coarse-to-fine profiling which significantly reduces computation time, slower than would be desired for any real-time application. We use NVIDIA K20 GPU to train our model. Average necrosis and non-necrosis pixels of an image for the challenge are 1,330,000 and 2,900,000 respectively. At test time, the average computation time for predicting segmentation of an entire image using our slide windows approach is second scale on this GPU.

**Table 3:** Classification performance using cross-validation in training data from the MICCAI challenge

	Accuracy
Hang Chang [30]	85.83%
Our method [19]	97.8%
Jocelyn Barker [57]	100.0%

Adding our colon dataset and multiclass classification scenario, we compare several methods on both the brain tumor and colon cancer datasets. The performances are summarized in Table 4. MCIL is excluded from the multiclass classification comparison due to the limitations of the algorithm. In all cases, our method (SVM-CNN) yields statistically significant results.

**Table 4:** Classification methods comparison

Dataset	MCIL	TRANS	SVM-IMG	SVM-MF	SVM-CNN
MICCAI brain	91.1%	86.7%	62.2%	77.8%	97.8%
CRC binary	95.5%	92.3%	94.3%	90.1%	98.0%
CRC multiclass	-	78.5%	79.0%	75.5%	87.2%

For brain tumor classification of the GBM and LGG subtype, CNN features are much more powerful than manual features (MF) and yields 20.0% improvement in performance. Compared with MCIL and TRANS, our proposed framework is 6.7% and 9.1% better, respectively.

For colon cancer binary classification, while our method yields the highest performance similar to the results in brain tumor, all methods achieve at least 90% accuracy. In the multiclass scenario, only our method achieves accuracy over 80%. Compared with other approaches, SVM-CNN beats SVM-IMG when using the full image directly by 8.2% and beats SVM-MF that uses hard-coded manual features by 11.6%. Surprisingly, in colon cancer, SVM-IMG performs better than SVM-MF by about 4%.

In binary classification, both MCIL and SVM-CNN achieve significantly better performance than other methods. Since MCIL is a multiple instance learning based algorithm, while our framework adopts the feature pooling technique, which is similar to multiple instance learning, the main performance difference is contributed by the powerful CNN feature. Using extracted features trained on a general image database enables us to capture complex and abstract patterns even if the number of training images is limited.

To better capture which features have been activated in our histopathology image analysis methods, the image-level heatmap (Figs. 5 and 6) and feature patch characteristic (Figs. 7 and 8) are plotted. They are discussed in Section 3.4.

### Segmentation results

In the MICCAI challenge, our final segmentation submission also achieves first place with an accuracy of 84% on testing data. Table 5 shows the top performances from other participating teams [28]. Our framework outperforms the second-place team by 11%.

**Table 5:** Segmentation performance in the MICCAI challenge

	Accuracy	Place
Anne Martel	63%	4th
Hang Chang	68%	3rd
Siyamalan Manivannan [58]	73%	2nd
Our method [19]	84%	1st

Table 6 summarizes the segmentation performance of various methods on both the brain tumor and colon cancer dataset. GraphRLM is not suitable for comparison here since it is an unsupervised method. For the brain tumor dataset, SVM-CNN shows a 21.0% improvement in performance over SVM-MF. Using fine-tuned CNN further improves SVM-CNN by 0.4%.

**Table 6:** Segmentation methods comparison

Dataset	GraphRLM^1^	SVM-MF	SVM-CNN	SVM-FT
MICCAI brain	-	64.0%	84.0%	84.4%
CRC	-	77.0%	93.2%	94.8%

GraphRLM is an unsupervised method

**Fig. 3:**
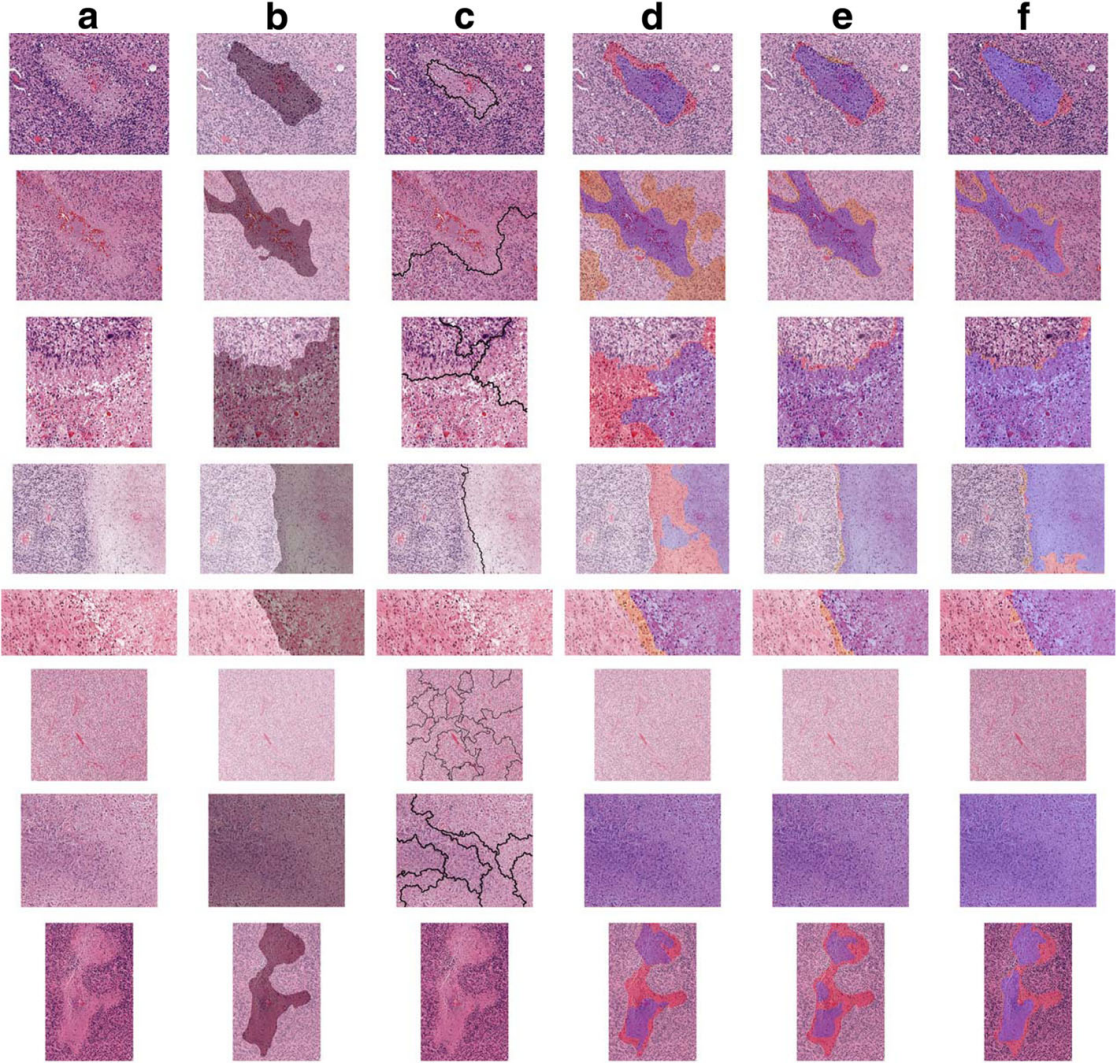
Segmentation results for the brain tumor dataset. **a** the original images. **b** ground truth of necrosis (positive) region masked *gray*. The rest of the columns show the prediction results by **c** GraphRLM, **d** SVM-MF, **e** SVM-CNN, and **f** SVM-FT methods where true positive, false positive (missed), and false negative (wrongly predicted) region are masked *purple*, *pale red*, and *orange*, respectively

**Fig. 4:**
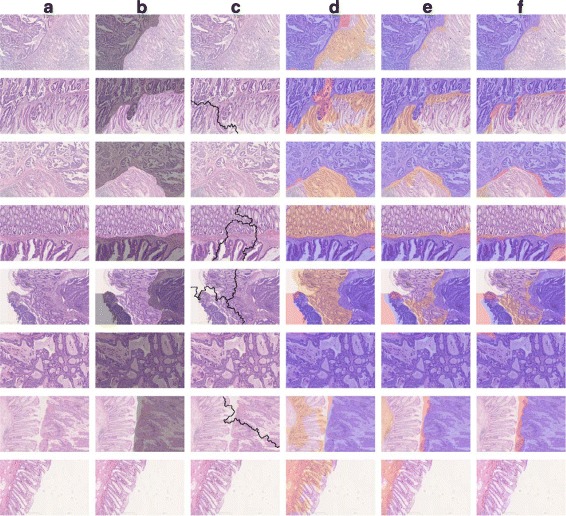
Segmentation method comparison for the colon cancer dataset. **a** the original images. **b** ground truth of necrosis (positive) region masked gray. The rest of the columns show the prediction results of **c** GraphRLM, **d** SVM-MF, **e** SVM-CNN, and **f** SVM-FT methods where true positive, false positive (missed), and false negative (wrongly predicted) region are masked *purple*, *pale red*, and *orange*, respectively

For colon cancer, CNN-based methods show at least 16.2% performance improvement over SVM-MF, so the results indicate a similar trend with the brain cancer dataset. After fine-tuning, accuracy further increases to 94.8%, showing a 1.6% difference. In addition, we provide some samples of the segmentation results using all methods, shown in Figs. 3 and 4 for the brain tumor and colon cancer dataset, respectively.

From Table 6, a significant performance difference can be observed using CNN-based features rather than manual hard-coded features. Using fine-tuned CNN features improves the accuracy of CNN features by 1% in colon cancer. The difference can also be verified by both Figs. 3 and 4. For GraphRLM, the segmentation results are incomprehensible or no segmentation result is provided. Although the result of GraphRLM cannot be precisely quantified, it fails to outline valuable boundaries or generates no boundary in most cases. Even in colon cancer, the same cancer type used in their publication, GraphRLM cannot provide segmentations that share similar morphological patterns. On the other hand, all other methods achieve at least 64% accuracy. SVM-CNN and SVM-FT show discernible improvement in performance over SVM-MF both in accuracy statistics and visualization.

### Selection of patch size

In our classification framework, the size of sampled patches is 336 ×336 pixels for 20 × magnification and 672 ×672 pixels for 40 × magnification scale. We also try other patch sizes to explore the influences of different patch sizes. Results are shown in Table 7. From the results in Table 7, we find that a patch size of 672 ×672 yields the highest accuracy on both binary and multiclass classification tasks.

**Table 7:** Classification results of different patch sizes

Dataset	224 ×224	448 ×448	672 ×672
MICCAI brain	91.1%	93.3%	97.8%
CRC binary	97.5%	96.9%	98.0%
CRC multiclass	85.0%	85.3%	87.2%

Patch sizes in the table correspond to 40 × magnification scale. For 20 × magnification scale, the sizes are halved

In our segmentation framework, a patch size of 112 ×112 pixels is chosen. We also explore the influences of patch size on our segmentation framework. The results are shown in Table 8. From the results, it shows that a smaller patch size will give rise to better segmentation results on both datasets. This fact follows our intuitions. In the segmentation framework, labels of positive or negative are given to each sampled training patch based on its overlapping ratio with annotated region, and segmentation result is constructed from predicted labels of all sampled patches. In this condition, larger patch size will affect the resolution of the boundary of the segmented region, which hurts the accuracy of the segmentation results.

**Table 8:** Segmentation results of different patch sizes

Dataset	112 ×112	224 ×224	336 ×336
MICCAI brain	84.0%	78.5%	75.7%
CRC	93.2%	86.9%	81.3%

### Visualization of CNN activation features

Our proposed frameworks adopting CNN features show high accuracy on both the brain tumor and colon cancer dataset. We are interested in what exactly our classifiers have learned from CNN features and whether they can reveal biological insights. For this purpose, individual components of the responses of neurons in the last hidden layer (4096 dimensions) are visualized to observe the properties of CNN features. In particular, we visualize their image-wise and feature-wise responses to understand which part of the image our CNN finds important.

**Fig. 5:**
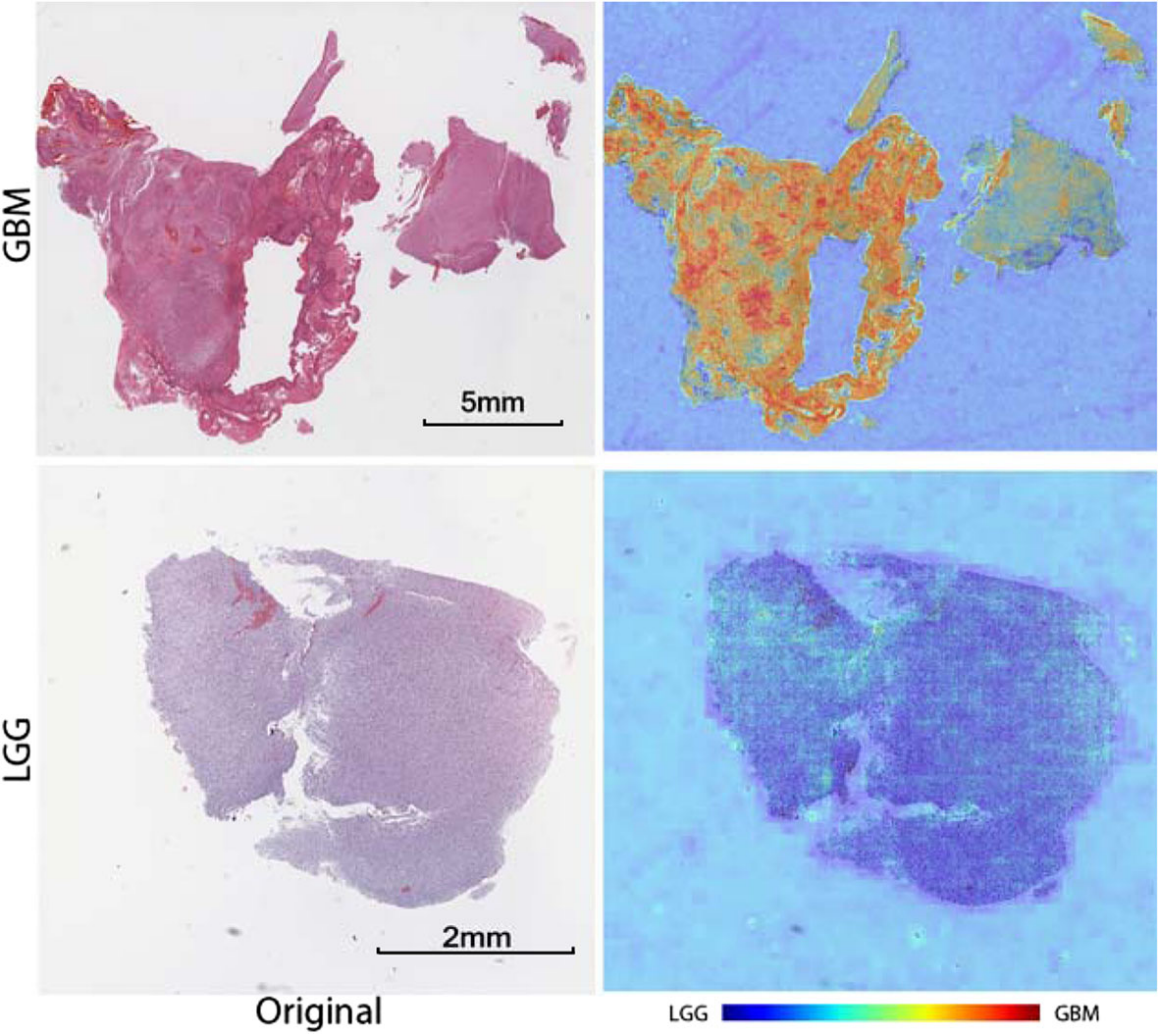
Heatmap for brain tumor GBM vs LGG classification. Each patch of the whole slide image is assigned a confidence using the classifier, which forms the heatmap. Regions that are red in color are more likely to be GBM regions. The purpose of these heatmaps is to illustrate which part of the whole slide image is considered important for the classifier and to prove the expressiveness of CNN features. In the GBM example, the endothelial proliferation regions, which are considered an essential morphologic cue for the diagnosis of GBM, show high positive confidence

**Fig. 6:**
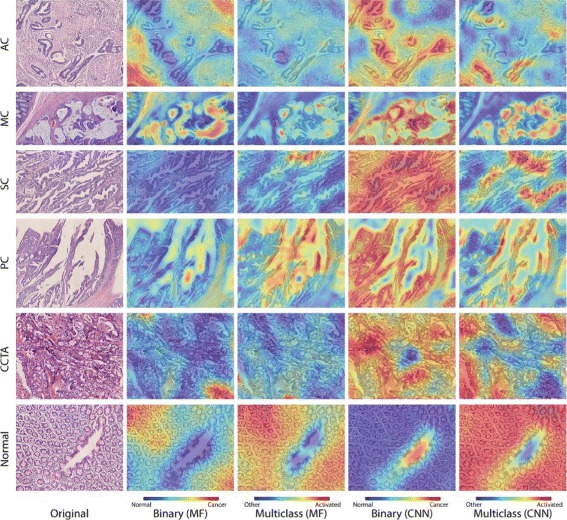
Heatmap for binary and multiclass classification of colon cancer using both manual features and CNN activation features. Similar to Fig. 5, heatmap is drawn based on confidence scores of each patch, and the purpose is also to explore the expressiveness of CNN features. In binary classification (2nd and 4th column), red regions are more likely to be cancer. In multiclass classification (3rd and 5th column), only the classifier that predicts the image’s label is shown, that is, for the AC image, only the prediction of the AC-vs-rest classifier is shown. Areas that are red are more likely to be the image’s label. The transition of the highlighted regions from binary to multiclass classification indicates that our multiclass classifiers can recognize the specific characteristics of each cancer subtype. The comparison between the CNN features and manual features shows the CNN features have greater power of expressiveness than the manual features

From the aspect of images, each patch is assigned a confidence using the classification model trained by linear SVM. We visualize the confidence score of each patch as a heatmap (Figs. 5 and 6). The redder (resp. blue) a region is, the more confident the classifier will be to consider that region being positive (resp. negative). Heatmaps help to visualize the important regions the classifier thinks. For each classification task, one image from each category is shown in the paper.

**Fig. 7:**
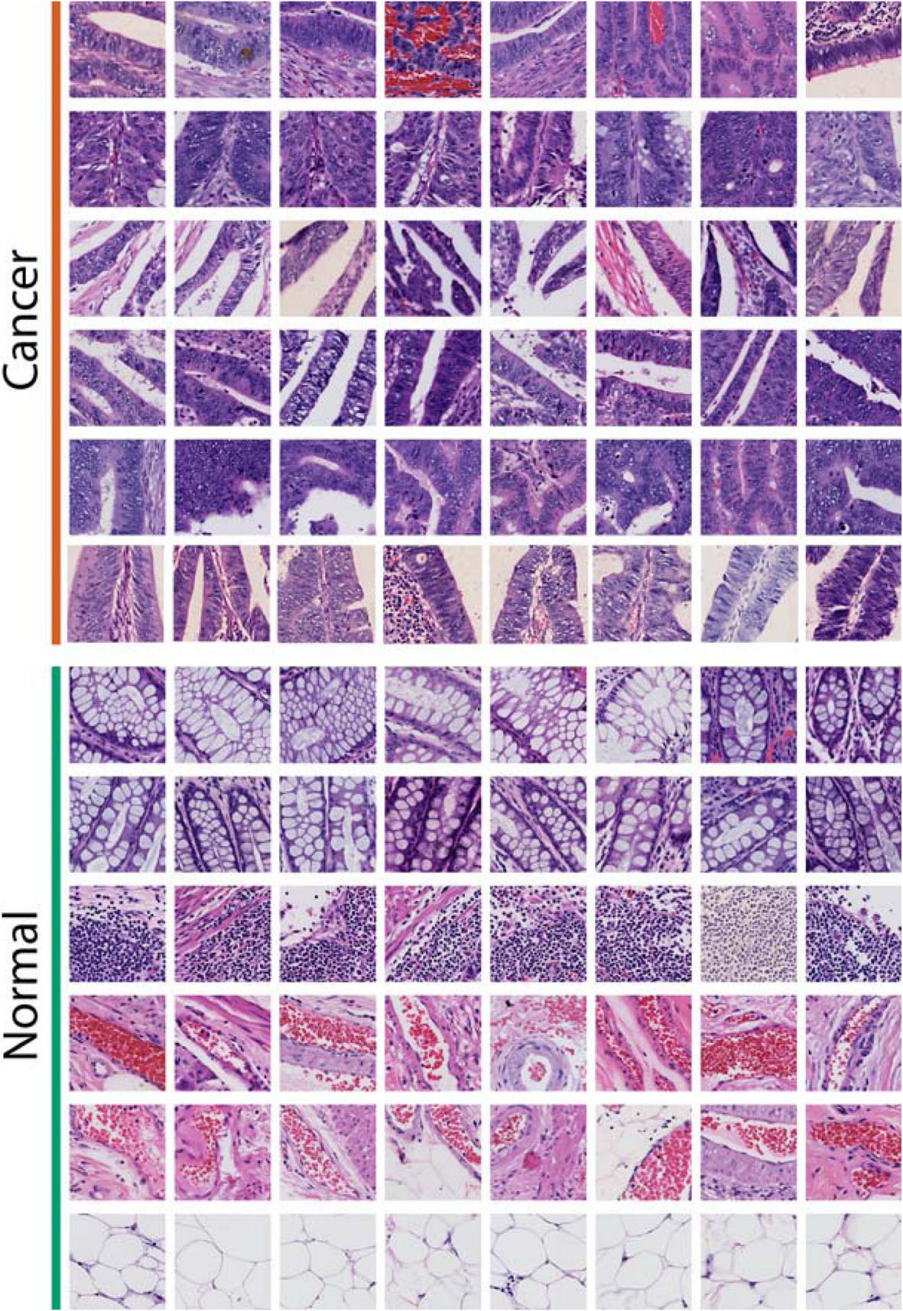
Sample discriminative patches selected with individual components (neurons) of the CNN activation features. Each row of patches causes a high response in one of the 4096 neurons from all colon training images in binary classification task. 6 top-weight features for each classifier are selected and top patches triggering these 6 neurons are selected to represent the characteristics of the corresponding feature. The purpose of this figure is to show the characteristics of individual components of CNN features which are thought be important by the binary classifier. These visualized characteristics convey some clinical insights

**Fig. 8:**
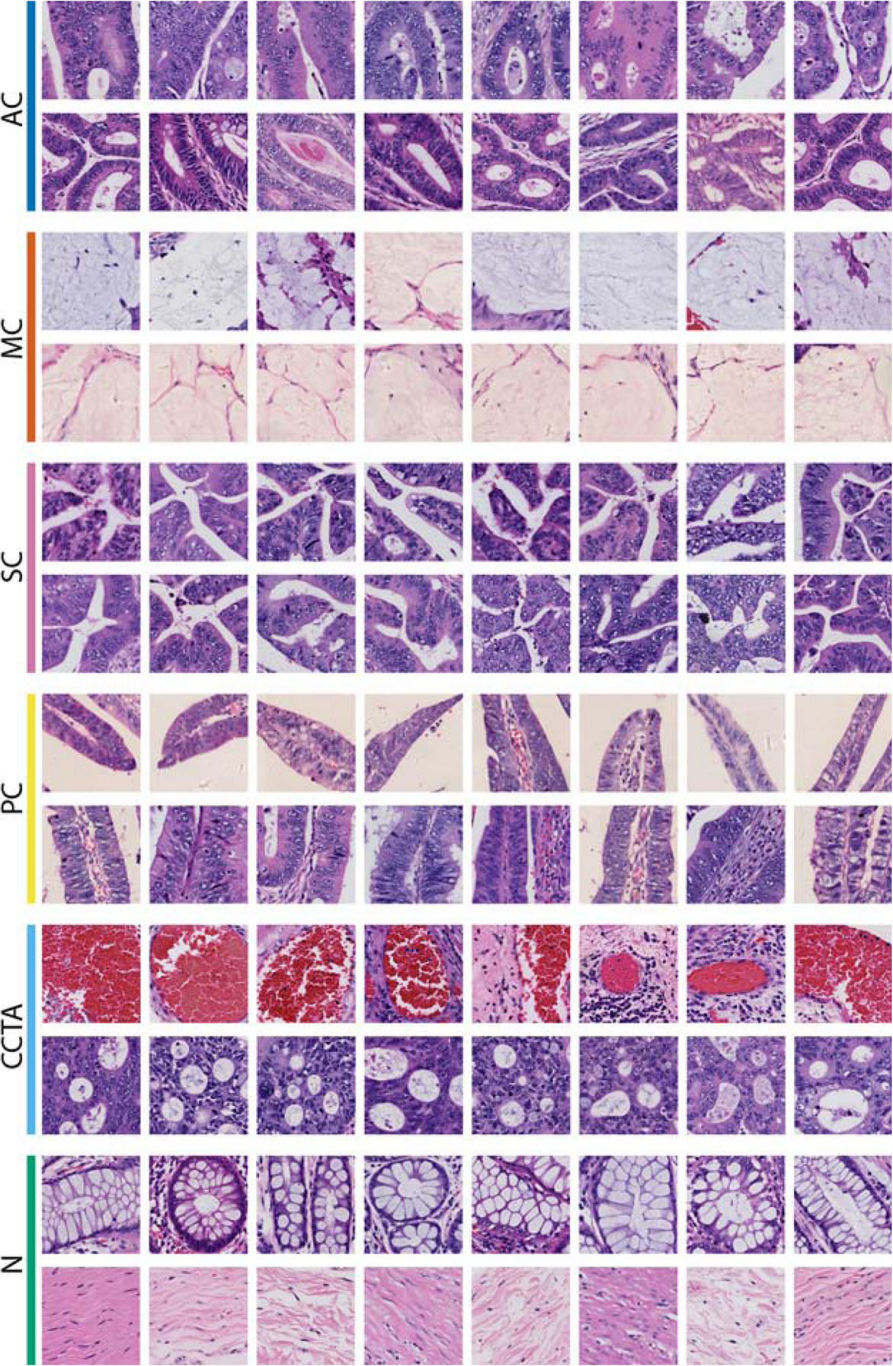
Sample discriminative patches selected with individual components (neurons) of the CNN activation features. Each row of patches causes a high response in one of the 4096 neurons from all colon training images in multiclass classification task. Two top-weight features for each classifier are selected and top patches triggering these two neurons are selected to represent the characteristics of the corresponding feature. The purpose of this figure is to show the characteristics of individual components of CNN features which are thought be important by the multiclass classifier. These visualized characteristics convey some clinical insights

In terms of features, we visualize the response of individual neurons in the last hidden layer to observe the characteristics of CNN features (Figs. 7 and 8). The top activated feature dimensions are determined by the highest weights from the classification SVM model. For the relevant neurons, patches that activate them the most are selected (patches that have highest value in that feature dimension).

#### Image-level heatmaps

Though we do not explicitly label the attributes of each cancer type, the heatmaps of our classifiers show they indeed highlight the representative hot spots. For example, necrosis regions, which are characteristics of GBM, are generally considered highly positive.

For brain tumors, heatmaps are shown in Fig. 5. We have the whole of all slide images labeled as GBM and LGG, respectively. In this classification scenario, both classes are glioma, but with different glioma grades. High grade glioma includes anaplastic astrocytomas and glioblastoma multiforme, which come with the presence of necrotic regions and hyperplastic blood vessels and megakaryocytes and are detectable using an H&E stain. In the example of heatmaps, the endothelial proliferation regions of GBM are well captured.

For colon cancer, heatmaps for both binary and multiclass classification are shown in Fig. 6. In the binary scenario, our CNN successfully recognizes the malformed epithelial cells in cancer instances and evenly spaced cell structure in normal instances. For example, in the example of the adenocarcinoma (AC) subtype, most of the malignant ductal elements shown in the figure are highlighted by the binary classifier. For the rest of the image, stromal cells are abundant and considered neutral or normal, as they are biologically benign. The lumen part shown in the normal example is misclassified as a cancer-like region since it resembles the shape of ill-shaped epithelial cells. However, some specific features of each cancer subtype are overlooked by the binary classifier. In the mucinous carcinoma (MC) example, the classifier recognizes the dense epithelium but ignores the primary characteristic of MC, where abundant extracellular mucin (light purple region in original image) can be seen. This is due to the similarity between the colloid and empty areas, which makes it more difficult to discern in the binary scenario.

In the multiclass scenario, specific characteristics for each subtype are stressed and become obvious in their classifier heatmap. In the MC example, only the colloid part triggers the MC classifier and other malignant parts are suppressed. The unique patterns of serrated carcinoma (SC) and papillary carcinoma (PC) are successfully captured by their classifier. In the SC subtype, different from the situation all regions are recognized as malignant, only the tooth-like epithelial structure remains highly confident. In the PC subtype, only the elongated tubular structure is highlighted. Many unique SC patterns are ignored by the classifier since they resemble the tubular characteristic of PC under our patch scale. For cribriform comedo-type adenocarcinoma (CCTA), its distinct cribriform characteristic that exhibits frequent perforation is highlighted in the heatmap. For the AC subtype, many malignant ductal elements are disregarded by the classifier from the binary to multiclass scenario, which is due to the similar ubiquitous structures in all cancer subtypes which are not helpful for improving the performance. For the normal example, the binary and multiclass classifiers show consistent results, while in the multiclass the lumen part in the middle of the image is correctly suppressed.

To compare CNN activation features with other features, heatmaps of manual features are also shown in Fig. 6. From the figures, we can clearly see the advantage of CNN activation features.

#### Feature patch characteristic

In the CNN features extracted from distinct medical images, we find that single feature dimension can indicate certain characteristic, which is one of the exciting discoveries made when applying visualization of CNN activation features. Even though there might exist certain types of manually designed features providing the same characteristics, CNN is able to learn these characteristics from large image dataset automatically, without any manual designs. Reported by histopathologists, some of the features can convey clinical insights, which can also verify our finding from the image-level heatmap analysis. The characteristics of each feature are visualized by selecting patches from all the images with the highest weights. For more details on the brain tumor images, we refer the reader to [19].

For colon cancer, the most discriminative features in both binary and multiclass classification are visualized, and shown in Figs. 7 and 8 respectively. Similar to the finding in heatmaps, even though we did not supply extra information about any pathological characteristic, features with high weights in a classifier corresponds to specific characteristics of a category. In binary classification, important cancer features include glandular cancer (1st, 2nd, 4th, 5th and 6th row), and papillary shapes (3rd row); while important non-cancer features include normal glands (1st and 2nd row), lymph cell clusters (3rd row), hemorrhage (4th and 5th row), and fat (6th row).

The multiclass classifier automatically discovers features more specific to each of the subtypes, with some cases particularly interesting and potentially instructive. For example, CCTA features not only contain the aforementioned cribriform structure (2nd row) as expected, but also contain a feature activated on hemorrhage regions (1st row) — suggesting some undiscovered correlation between CCTA and hemorrhage.

Many CNN features also suggest some new criteria for classifying cancer tissues. For example, PC features distinguish the tip (1st row) and middle part (2nd row) of its special tubular structure. MC features seem to separate patches of colloid secretion by the density of mucus: the first row of patches has more mucus than the second row. The two features visualized here for AC look very similar (both showing the dense epithelial lining of the colon duct), and the same can be recognized as glandular structures. In CCTA patch characteristics, features of the aforementioned cribriform structure (1st row) and hemorrhage (2nd row) are turned on, both being typical characteristics of CCTA. Noting that even though patches of hemorrhage shown here do not belong to the properties of colon cancer, they can still represent a neuron in the last CNN hidden layer that is often triggered by the features of the hemorrhage. For the normal type, features containing patches of longitudinal and transverse crypts (intestinal gland, 1st row) or patches of stroma cells (2nd row) are turned on.

## Conclusions

In this paper, we introduce deep convolutional activation features trained with ImageNet knowledge and apply a CNN model to the extraction of features from brain tumor and colon cancer digital histopathology datasets. We successfully transfer ImageNet knowledge as deep convolutional activation features to the classification and segmentation of histopathology images with relatively little training data. According to our experiments, CNN features are significantly superior to manual features. Additionally, due to the vast size of a single histopathology image, feature pooling technique is adopted to construct the single image-level feature vector in our classification framework. Experiments demonstrate that our frameworks achieve state-of-the-art results of 97.5% for classification and 84% for segmentation in the MICCAI brain tumor challenge. Later, we apply both frameworks on colon cancer images and achieve similar success, showing remarkable improvement over previous methods.

Moreover, the features learned by our classifier yield biologically meaningful insights that are recognized by pathologists. Jointly, the histopathology morphology from these selected patches or regions will help pathologists discover patterns with biological insight. By observing the discriminative patches with the individual neurons of CNN activation features, we can discover tissue components of corresponding subtypes. It is useful to explore the development process across different cancer stages and subtypes. By applying digital histopathology image analysis, subtle differences in complex morphology patterns can be captured and quantified, and we can re-investigate their joint interaction to reflect the prognosis or medicine response of patients and provide fine grained characterizations.

Our motivation is to introduce a general-purpose solution to histopathology problems. This makes our setup considerably simpler than most others. Fully convolutional networks (FCN) [18] are not suitable to classify large scale images. Therefore, we do not compare our method with FCN. In future work, we will compare our method with FCN in terms of segmentation.

## Abbreviations

AC: Adenocarcinoma
CNN: Convolutional neural network
CCTA: Cribriform comedo-type adenocarcinoma
GBM: Glioblastoma multiforme
KIRC: Clear cell kidney carcinoma
LGG: Low grade glioma
MC: Mucinous carcinoma
MCIL: Multiple clustered instance learning
MF: Manual features N: Normal
PC: Papillary carcinoma
RBM: Restricted boltzmann machine
SC: Serrated carcinoma
SVM: Support vector machine
VLAD: Vector of locally aggregated descriptions
